# Improving Cell Engraftment in Cardiac Stem Cell Therapy

**DOI:** 10.1155/2016/7168797

**Published:** 2015-12-13

**Authors:** Xiaofei Li, Kenichi Tamama, Xiaoyun Xie, Jianjun Guan

**Affiliations:** ^1^Department of Materials Science and Engineering, The Ohio State University, 2041 College Road, Columbus, OH 43210, USA; ^2^Department of Pathology, University of Pittsburgh School of Medicine, 3550 Terrace Street, Pittsburgh, PA 15261, USA; ^3^McGowan Institute for Regenerative Medicine, University of Pittsburgh, Pittsburgh, PA 15219, USA; ^4^Department of Gerontology, Tongji Hospital, Tongji University, Shanghai 200065, China; ^5^Tongji Hospital, Tongji University, Shanghai 200065, China

## Abstract

Myocardial infarction (MI) affects millions of people worldwide. MI causes massive cardiac cell death and heart function decrease. However, heart tissue cannot effectively regenerate by itself. While stem cell therapy has been considered an effective approach for regeneration, the efficacy of cardiac stem cell therapy remains low due to inferior cell engraftment in the infarcted region. This is mainly a result of low cell retention in the tissue and poor cell survival under ischemic, immune rejection and inflammatory conditions. Various approaches have been explored to improve cell engraftment: increase of cell retention using biomaterials as cell carriers; augmentation of cell survival under ischemic conditions by preconditioning cells, genetic modification of cells, and controlled release of growth factors and oxygen; and enhancement of cell survival by protecting cells from excessive inflammation and immune surveillance. In this paper, we review current progress, advantages, disadvantages, and potential solutions of these approaches.

## 1. Introduction

Heart disease has a high rate of morbidity and mortality [1]. Myocardial infarction (MI) is a major heart disease that causes massive cardiac cell death and partial loss of heart function. The infarcted heart tissue cannot effectively regenerate by itself because adult cardiomyocytes are unable to proliferate, and cardiac stem cells spontaneously generate only a limited number of cardiomyocytes [2]. Heart function thus cannot be restored. Following MI, the left ventricular wall progressively becomes thinner, and heart function gradually decreases. This adverse remodeling process leads to heart failure [3]. Heart transplantation is the only solution for patients with end-stage heart failure, but the number of donors available for transplantation is extremely limited, and the recipients require long-term immune suppressants to prevent organ rejection. Stem cell therapy is an alternate strategy. It aims to regenerate the infarcted heart tissue and/or improve heart function.

## 2. Stem Cells for Cardiac Therapy

Multiple cell types have been tested in animal models and clinical trials for cardiac therapy. Some stem cell types are capable of differentiating into cardiomyocytes to regenerate the heart tissue, leading to the restoration of heart function. These cells include cardiac stem cells [4–8] and pluripotent stem cell-derived cardiovascular progenitor cells [9, 10]. Some stem cell types cannot differentiate into functional cardiomyocytes but provide paracrine effects to augment the survival of resident cardiac cells, vascularize infarcted heart tissue, modulate immune response, recruit endogenous stem cells, and facilitate beneficial remodeling [11–17], resulting in an overall improvement of heart function. These stem cells include bone marrow-derived stem cells [18–23], adipose-derived stem cells [24–27], and cardiosphere-derived cells (CDCs) [28–35].

In the majority of current animal studies and clinical trials, stem cells are injected directly into the infarcted heart. However approximately 90% of cells are lost to the circulation, leaked, or squeezed out of the injection site [36]. For those cells retained in the infarcted tissue, most of them die within the first few weeks [37]. Overall, cell engraftment of current stem cell therapy is low, and its therapeutic efficacy is limited.

## 3. Major Causes of Low Cell Engraftment in Infarcted Hearts

As discussed above, the major causes of the low cell engraftment are inferior cell retention and survival in the infarcted heart tissue. The commonly used saline solution has very low viscosity and cannot efficiently hold the cells in tissue. Transplanted cell death is mainly a result of inadequate cell attachment to the host tissue, severe ischemia, and excessive inflammation. Anoikis is a form of programmed cell death of adherent cells induced by poor or weak interaction between cell and extracellular matrix (ECM) [38]. In normal heart tissue, adherent cells attach strongly to the surrounding ECM. In the infarcted tissue, however, the ECM does not allow strong cell attachment [39]. Moreover, the saline used for cell transplantation does not provide cells with a matrix for attachment. These events cause anoikis [40].

Another factor is oxygen tension in the tissue. After MI, an extremely low oxygen and nutrient ischemic environment exists in the infarcted region. Although hypoxia is considered necessary to preserve the stem cell properties [41], the harsh ischemic environment activates cell death pathways, resulting in death of the transplanted cells [42].

Following MI, acute inflammation ensues with recruitment of inflammatory cells (neutrophils and monocytes) into the infarcted heart tissue. These recruited inflammatory cells are engaged in production of various inflammatory cytokines and chemokines to recruit more inflammatory cells, secretion of various proteolytic enzymes and reactive oxygen species (ROS), and phagocytosis to remove dead cells and tissue debris [43–45]. Both ROS and proinflammatory cytokines, such as tumor necrosis factor-*α* (TNF-*α*), can compromise survival of transplanted cells.

## 4. Approaches to Improve Stem Cell Engraftment

**Figure 1:**
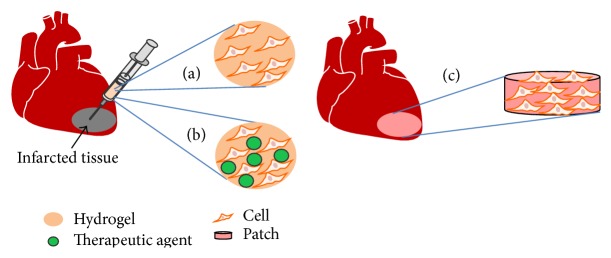
Strategies to improve cell retention in infarcted hearts. An injectable hydrogel can be used as a delivery vehicle for cells (a), or cells and therapeutic agents such as genes or proteins (b). A scaffold can be seeded with cells* in vitro* and then implanted to the infarcted region (c).

To increase cell engraftment in infarcted hearts, improving both cell retention and survival is necessary. The former may be achieved by using viscous, injectable hydrogels as cell carriers since low viscosity saline cannot efficiently hold cells in tissue. An injectable hydrogel can be delivered into the infarcted hearts through a minimal invasive injection approach (Figure 1) [49]. Seeding cells into scaffolds and patching them onto the heart may also increase cell retention (Figure 1). Both injectable hydrogels and scaffolds can augment cell survival. They provide an environment for cell attachment, which is required for cell survival. They can also be modified to promote stem cell survival under ischemic and inflammatory conditions [50]. In addition, the hydrogels and scaffolds offer mechanical support to the infarcted tissue to improve cardiac function.

To address the issue of cell survival under ischemic conditions, approaches including ischemic preexposure of cells, genetic modulation of cells, and delivery of growth factors and oxygen to cells have been used. To promote cell survival under inflammatory conditions, biomaterials have been modified to prevent immune proteins and proinflammatory cytokines from penetrating inside to attack the encapsulated stem cells.

### 4.1. Using Biomaterials and Cell Adhesion Molecules for Stem Cell Delivery

Biomaterials used for stem cell transplantation should be biodegradable and biocompatible [51]. Specifically, they should have a controlled biodegradation rate, which ideally coincides with the rate of new tissue regeneration [52]. The degradation products should be nontoxic. The biomaterials should ideally mimic mechanical properties of the heart tissue, for example, stiffness. This will decrease the elevated wall stress to improve cardiac function [53]. Both natural and synthetic polymers have been employed for stem cell transplantation. Natural polymers are biologically derived materials. Some of them, like fibrin [49, 54], alginate [55–57], collagen [58, 59], Matrigel [60], hyaluronic acid [61], and chitosan [62], have been used to deliver stem cells into infarcted hearts.

Synthetic polymers are generated via chemical method to pursue desired properties and functions. The properties can be controlled by composition and chemistry. The capability of endowing synthetic polymers with functional groups and tunable properties are advantages of using these polymers for stem cell transplantation [63]. Commonly used synthetic polymers include polyesters, such as polyglycolide (PGA), polylactide (PLA), poly(lactide-co-glycolide) (PLGA), polycaprolactone (PCL), and their copolymers [64]. These polymers are often used in the form of scaffold. PLGA scaffolds loaded with bFGF have been used to promote cardiac angiogenesis [65]. Porous PCL scaffolds have been used to deliver endothelial progenitor cells into heart tissue to promote vascularization [66].

**Figure 2:**
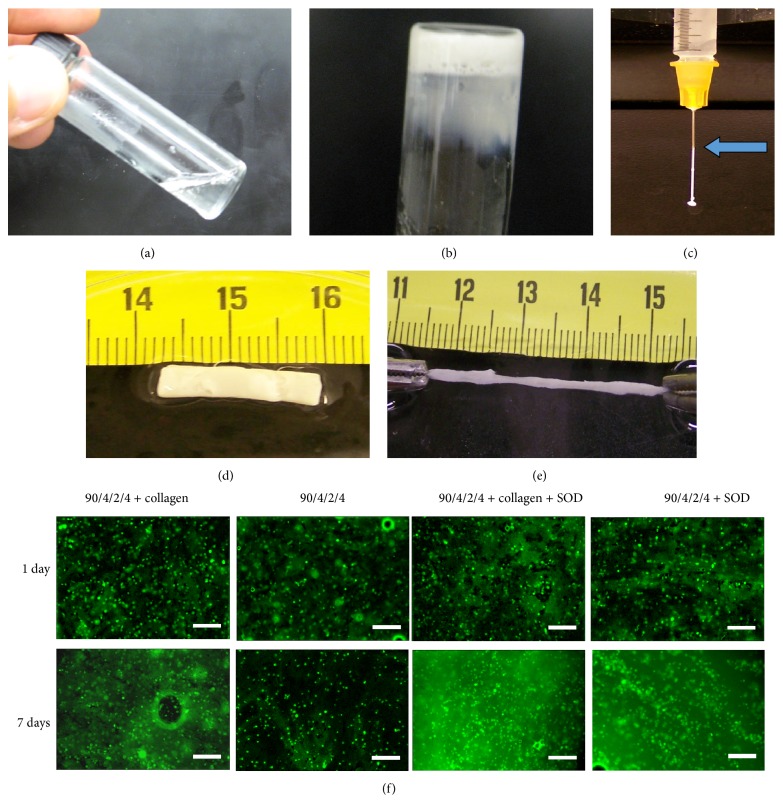
Macroscopic images of hydrogel and cells. The copolymer solution is flowable at 4°C (a) and forms gel after gelation at 37°C (b). The copolymer solution can be injected through a 26-gauge needle (c). At 37°C, the formed gel is flexible and can be stretched: (d) before stretching; (e) after stretching; (f) fluorescence images of MSCs encapsulated in hydrogels with or without collagen and superoxide dismutase (SOD, 4 mg/mL) after 1 and 7 days of culture. The cells were stained with live cell stain CMFDA before encapsulation. Scale bar = 100 *μ*m. This figure is adopted from [46].

Some synthetic polymers can be used in the form of hydrogel. For example, Li et al. generated thermosensitive hydrogels based on N-isopropylacrylamide (NIPAAm), acrylic acid (AAc), dimethyl-gamma-butyrolactone acrylate (DBA), and 2-hydroxyethyl methacrylate-poly(trimethylene carbonate) (HEMAPTMC) [46]. The hydrogels are injectable at room temperature and solidify at body temperature within 10 seconds. They can therefore quickly solidify to efficiently hold cells in the tissue. Interestingly, mesenchymal stem cells (MSCs) were able to proliferate inside (Figure 2). Other synthetic hydrogels developed for cardiac repair include poly(ethylene oxide)-b-poly(propylene oxide)-b-poly(ethylene oxide) (PEO-PPO-PEO) [67], poly(D-lysine) (PDL) [68], and MPEG-PCL-MPEG [69, 70].

Biomaterials can be modified with cell adhesive molecules to improve cell attachment, thus decreasing anoikis-induced stem cell death during transplantation [71]. Cell adhesive molecules are often mixed with or conjugated to the biomaterials. Karoubi et al. studied MSC survival in agarose with and without the addition of fibronectin and fibrinogen [72]. The results showed that cell survival was significantly increased after addition of fibronectin and fibrinogen. Similarly, fibrin glue remarkably improved cell survival in infarcted hearts [54]. Cooke et al. investigated cell adhesion on surfaces modified with several cell adhesive molecules, collagen I, collagen IV, fibronectin, and laminin, and found that cell attachment was increased [71]. Peptides YIGSR/IKVAV and RGD derived from laminin and fibronectin, respectively, have also been used to modify biomaterials to improve cell affinity [73].

### 4.2. Preexposure of Stem Cells for Enhanced Cell Survival

Preexposure of stem cells with ischemia or cytokines for cytoprotection is an alternate strategy to alleviate cell death. It enhances the cell tolerance to the harsh ischemic conditions. Murry et al. showed that cyclic exposure of stem cells to ischemia improved cell survival in ischemic myocardium [74]. Maulik et al. further demonstrated that ischemic preexposure allowed cells to adapt to ischemia, thus attenuating cell death under ischemia [75]. Grund et al. found that ischemically preexposed cells had reduced oxygen consumption [76]. In addition, ischemic preexposure enhanced cell secretion of growth factors [77, 78].

Cytokine preexposure can also improve cell survival [79–81]. MSCs pretreated with SDF-1*α* released antiapoptotic and angiogenic cytokines to improve cell survival and augment tissue angiogenesis [79]. Preexposure of endothelial progenitor cells with VEGF and bFGF enhanced cell paracrine effects, resulting in enhanced cell survival, angiogenesis, and reduced infarct size and left ventricle remodeling [80, 81]. Cells pretreated with IGF-1 showed cytoprotection effect both* in vitro* and* in vivo* [82, 83]. PI3K/Akt and MAPK/Erk1/2 pathways are responsible for the prosurvival effect.

### 4.3. Release of Growth Factors to Improve Cell Survival

High rates of both short-term and long-term cell survival are necessary for cardiac stem cell therapy. These may be achieved by using growth factors. Prosurvival growth factors allow for short-term cell survival, while angiogenic growth factors stimulate angiogenesis for long-term cell survival [84–86]. IGF-1 and HGF are two commonly used prosurvival growth factors. FGF [87], PDGF [88], and VEGF [89] are angiogenic growth factors used to promote angiogenesis in various tissues. bFGF also enhances cell survival under ischemic conditions [47]. In addition, specific growth factors can be used with prosurvival and angiogenic growth factors to promote stem cell differentiation into functional cells such as cardiomyocytes [90–92].

However, a concern for using growth factors in stem cell transplantation is that most of them have a relatively short half-life [93, 94]. Genetic modification of stem cells to promote the cells to secrete prosurvival and proangiogenic growth factors and sustained release of growth factors using biomaterials are commonly used approaches to address this concern.

Stem cells transfected with encoded genes of angiogenic growth factors like VEGF and FGF, and antiapoptotic factors like Akt and heme oxygenase-1, were able to secrete autocrine and paracrine growth factors [95, 96]. After transplanting these cells into infarcted hearts, cell survival, angiogenesis, and heart function were improved [97–99]. Matsumoto et al. transfected VEGF gene to MSCs and injected the modified cells into MI rat hearts [100]. High expression of VEGF was detected. This not only reduced cell death and increased capillary density, but also decreased the infarct size.

**Figure 3:**
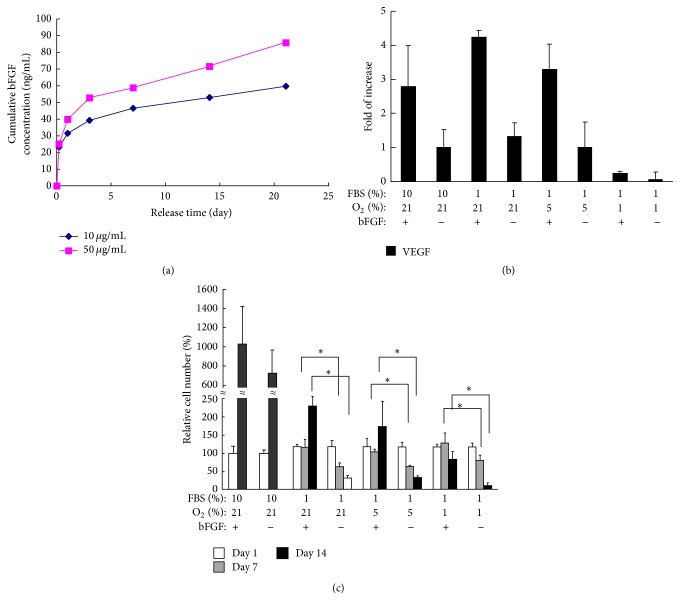
(a) Release kinetics of bFGF loaded in the hydrogels. bFGF loading was 10 and 50 *μ*g/mL, respectively. The error bars are small. (b) VEGF expression of MSCs in the hydrogels under different culture conditions. Cells were cultured under conditions of 10% FBS and 21% oxygen, 1% FBS and 21% oxygen, 1% FBS and 5% oxygen, and 1% FBS and 1% oxygen, respectively. The expression was normalized by that under 10% FBS with 21% oxygen culture condition. (c) MSC survival in hydrogels cultured under different conditions. Culture conditions: 10% FBS and 21% oxygen, 1% FBS and 21% oxygen, 1% FBS and 5% oxygen, and 1% FBS and 1% oxygen. Double stranded DNA (dsDNA) content was used to quantify live cell number in the hydrogels. The dsDNA content at day 1 (100%) was used for normalization. This figure is adopted from [47].

The growth factors are often encapsulated in biomaterials for controlled release [101–103]. Li et al. developed a bFGF release system based on a thermoresponsive and degradable hydrogel [47]. bFGF can be gradually released from the hydrogel for more than 2 weeks. The bFGF releasing system significantly increased MSC survival under low oxygen and nutrient conditions (Figure 3). It is expected that transplantation of stem cells using this bFGF release system will augment cell survival in the infarcted hearts. The release system may also promote angiogenesis due to the angiogenic effect of bFGF.

Padin-Iruega et al. tethered IGF-1 to self-assembling peptide nanofibers and used them for delivery of cardiac progenitor cells (CPCs) into infarcted hearts [104]. The IGF-1 was found to continuously release from the nanofibers to the myocardium. The released IGF-1 not only augmented CPC survival but also promoted cardiac differentiation, leading to enhanced cardiac regeneration.

### 4.4. Augmentation of Cell Survival by Releasing Oxygen to Transplanted Cells

Oxygen is critical for cell survival. The extremely low oxygen concentration in the infarcted heart results in significant cell death [105, 106]. Transplantation of stem cells with an oxygen release system is considered a feasible strategy to improve cell survival [107].

An oxygen release system can be generated using inorganic peroxide. Oh et al. developed a calcium peroxide-based oxygen release system by incorporating calcium peroxide into PLGA scaffold [108]. The system can continuously release oxygen for 10 days. The released oxygen enhanced cell survival under hypoxic conditions* in vitro*. However, this approach is not well suited for cardiac application since the Ca^2+^ generated together with oxygen may lead to abnormal Ca^2+^ transient in cardiomyocytes [109]. To avoid the ion effect, organic molecules-based oxygen release systems, such as pyridine endoperoxide oxygen release system [110], hydrogen peroxide/poly(methyl methacrylate) microcapsule oxygen release system [111], and porphyrin-hemoprotein (rHSA(FeP-Glu)) oxygen release system [112], were developed and found to enhance cell survival. However, these oxygen release systems can release oxygen for less than 24 hours [108, 110–112].

**Figure 4:**
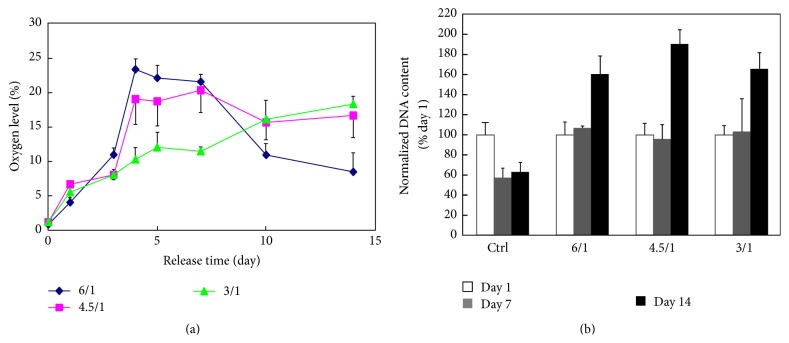
(a) Oxygen release kinetics of the H_2_O_2_ -releasing microspheres with different H_2_O_2_/VP ratio; (b) dsDNA content of live CDCs encapsulated in hydrogels with or without oxygen release. The higher dsDNA content represents higher live cell number. Cells were cultured under 1% oxygen condition. Hydrogels with oxygen release had microspheres with H_2_O_2_/VP ratio of 6/1, 4.5/1, and 3/1, respectively. This figure is adopted from [48].

To achieve longer term oxygen release, Abdi et al. encapsulated hydrogen peroxide into PLGA microspheres and obtained oxygen release for 7 days [113]. Li et al. encapsulated hydrogen peroxide/PVP complex in the PLGA microspheres and achieved sustained oxygen release at a relatively high oxygen level for 2 weeks (Figure 4) [48]. This oxygen release system significantly improved cell survival under hypoxic conditions* in vitro*. It has a great potential to augment cell survival in infarcted hearts for an extended period of time. Yet the concentration of released oxygen needs to be controlled so as not to overproduce ROS.

### 4.5. Modification of Biomaterials to Enhance Cell Survival under Immune Rejection and Inflammation Conditions

Immune rejection and excessive inflammation also decrease the survival rate of transplanted cells. Proinflammatory cytokines like TNF-*α* and IL-1 induce excessive inflammation and create a noxious microenvironment, in addition to causing apoptosis of the cells [114]. Optimization of biomaterial properties and introduction of anti-inflammatory molecules into biomaterials may provide protection for the transplanted cells. By controlling pore size of the biomaterials, transplanted cells can be immunoisolated, leading to better cell survival of the transplanted cells [115–119]. However, small cytotoxic molecules such as TNF-*α* and IL-1*β* can still diffuse into the biomaterials [120, 121]. To address this issue, approaches like increasing degree of cross-linking and matrix concentration were used. However, these approaches may impede nutrient transport to cells. An alternate approach is to modify the biomaterials with anti-inflammatory molecules. For example, anti-TNF-peptide WP9QY (YCWSQYLCY) may be conjugated into hydrogel to prevent TNF from penetrating inside [122].

After MI, ROS content in the failing heart is upregulated [123], which can be cytotoxic against the transplanted cells. To decrease the cytotoxic effects of ROS, Hume and Anseth incorporated superoxide dismutase mimetic (SODm) into PEG hydrogel. This largely protected cells from oxidative stress damage and improved cell survival [124].

## 5. Conclusions and Prospects

Stem cell therapy is considered a potent and promising approach for cardiac therapy. However, the efficacy is limited as only a small percentage of transplanted cells engrafted in the infarcted tissue. Low cell retention and inferior cell survival are mainly responsible for the limited cell engraftment. Hydrogels and scaffolds can be utilized to improve cell retention. Injectable hydrogels may be more convenient for cell delivery than scaffolds as they can be delivered by a minimally invasive injection approach. Injectable hydrogels increase cell retention because of their high viscosity. Yet, long gelation time may not allow the hydrogels to largely increase cell retention, as they may be squeezed out of heart tissue or washed into circulation before gelation. Some hydrogels require UV radiation, pH changing, or ion addition to solidify, which may cause potential harm to cells. Thermosensitive and biodegradable hydrogels that have a fast gelation rate (in seconds) may address this issue.

Different approaches have been explored to enhance cell survival in infarcted hearts. While they can improve cell survival to some extent, different types of stem cells may require dissimilar optimization approaches for preparation, activation, transplantation procedures, and maintenance* in vivo*. There are also disadvantages associated with these approaches. Ischemic preexposure may damage cells in the process, and the transplanted cells may not survive under ischemic conditions for a long enough period. Genetic modification of cells may raise safety concerns. Controlled release of growth factors and oxygen and immune protection appear to be more effective to promote cell survival. However, further studies on the long-term effect of these approaches on cell survival, functioning, and differentiation are needed.

From a clinical point of view, safety and efficacy are still paramount issues. More studies on animals are required in order to develop a reliable cell-biomaterial delivery system with long-term safety and efficacy. Biomaterial type, degradation product toxicity, dose, and timing must be well studied before clinical application.
